# Designable Electrical/Thermal Coordinated Dual‐Regulation Based on Liquid Metal Shape Memory Polymer Foam for Smart Switch

**DOI:** 10.1002/advs.202205428

**Published:** 2023-01-19

**Authors:** Ruoxi Zhao, Sibo Kang, Chao Wu, Zhongjun Cheng, Zhimin Xie, Yuyan Liu, Dongjie Zhang

**Affiliations:** ^1^ School of Chemistry and Chemical Engineering Harbin Institute of Technology Harbin 150001 P. R. China; ^2^ State Key Laboratory of Marine Coating Marine Chemical Research Institute Co., Ltd. Qingdao 266071 P. R. China; ^3^ National Key Laboratory of Science and Technology on Advanced Composites in Special Environments Harbin Institute of Technology Harbin 150080 P. R. China

**Keywords:** controllable electrical/thermal conductivity, liquid metal, shape memory foam, smart switch

## Abstract

Electronic components with tunable resistance, especially with synergistic regulation of thermal conductivity, play important roles in the fields of electronics, smart switch, soft robots, and so on. However, it is still a challenge to get the material with various resistance and thermal conductivity stably without lasting external force. Herein, a liquid metal shape memory polymer foam (LM‐SMF) is developed by loading electrically and thermally conductive liquid metal (LM) on deformable foam skeleton. Based on thermal response shape memory effect, the foam skeleton can be reversibly pressed, the process of which enables LM to transfer between connected and disconnected states. As a result, obtained LM‐SMF shows that the resistance stably changes from 0.8 Ω (conductor) to 200 MΩ (insulator), and the thermal conductivity difference is up to 4.71 times (0.108 to 0.509 W m^−1^ K^−1^), which indicates that LM‐SMF can achieve the electrical and thermal dual‐regulation. Moreover, LM‐SMF can be used as a designable self‐feedback/‐warning integrated smart switch or tunable infrared stealth switch. This work proposes a novel strategy to get the material with electrical–thermal coordinated dual‐regulation, which is possibly applied in intelligent heating system with real‐time monitoring function, electrothermal sensor in the future.

## Graphical abstract

A liquid metal shape memory polymer foam (LM‐SMF) is reported, which can achieve dual‐regulation of electrical/thermal conductivity based on unique capacities of LM and shape memory effect. LM‐SMF is used as a designable self‐feedback/‐warning integrated smart switch or tunable infrared stealth switch, and can also be applied as electrothermal sensor with real‐time monitoring function in the future.

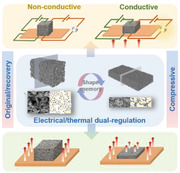

## 1 Introduction

With the rapid development of flexible electronics toward intelligence, complex electronic components, especially with variable resistance, play more and more important roles in the fields such as flexible electronic skins,^[^ ^1^ ^]^ soft robots,^[^ ^2^ ^]^ actuators,^[^ ^3^ ^]^ and sensors.^[^ ^4^ ^]^ Currently, a couple of strategies have been studied to fabricate the materials with convertible resistance, such as rigid metal–insulator transitions (such as perovskites,^[^ ^5^ ^]^ metal oxide,^[^ ^6^ ^]^ or organic film^[^ ^7^ ^]^), deep‐freezing temperature,^[^ ^8^, ^9^ ^]^ concentrated compressive/tensile stress,^[^ ^10^, ^11^, ^12^ ^]^ and controlling phase transition methods.^[^ ^13^ ^]^ For example, Zhang et al.^[^ ^14^ ^]^ designed a kind of stretchable phase‐transformable ionogel, which displayed alterable transition between insulator and conductor at a transition temperature ≈ −15 °C. Nevertheless, due to electrothermal effect and miniaturization of electronics, sometimes the circuit is short or overheated caused by current overload, and the other electronics in the system will be in danger,^[^ ^15^, ^16^ ^]^ meaning that the simple resistance controllable materials cannot satisfy the complex demand in real life. Therefore, it is significant to control resistance and thermal transmission of electronics at the same time.

Traditionally, researchers have attempted to add electrical and thermal conductive micro/nano fillers, containing carbon materials (carbon nanotubes^[^ ^17^ ^]^ and graphene^[^ ^18^, ^19^ ^]^), metal nanowires,^[^ ^20^ ^]^ inorganic materials,^[^ ^21^ ^]^ and so on, into polymer matrix to form an electrically/thermally conductive network. However, owing to the rigidity and high dispersion of micro/nano particles, the composites are hard to deform and produce continuous changing of electrical/thermal conductive network. Liquid metal (LM) is a promising material benefiting from its outstanding properties such as electrical and thermal conductivity, fluidity at low temperature, and biocompatibility,^[^ ^22^, ^23^, ^24^ ^]^ which can be constructed to various structure, including particles,^[^ ^25^, ^26^ ^]^ fibers,^[^ ^27^, ^28^ ^]^ foams,^[^ ^29^, ^30^ ^]^ and elastomer composites.^[^ ^31^, ^32^ ^]^ Based on the desirable properties of LM, the large‐range deformable LM elastomer composites (LMECs) have been obtained by adding LM into stretchable and flexible substrates,^[^ ^12^, ^33^, ^34^ ^]^ which can ensure the continuity of LM fillers in a relative high deformation range. To obtain LMECs, researchers have tried different methods, such as directly mixing LM particles into elastomer matrix (Ecoflex 00‐30,^[^ ^34^, ^35^, ^36^ ^]^ PDMS^[^ ^37^ ^]^ or other elastomer^[^ ^38^ ^]^), or injecting LM to elastomer foams prepared by template or foaming method.^[^ ^39^, ^40^ ^]^ Those LMECs have exhibited excellent electrical^[^ ^40^, ^41^ ^]^ or thermal adjusting,^[^ ^34^, ^42^ ^]^ but there are only a few works that can realize the electrical and thermal conductivity simultaneously so far. For example, Li et al.^[^ ^42^ ^]^ reported a LM‐filled magnetorheological elastomer with magnetic field‐responsive deformation, which shows tunable electrical and thermal properties under stretching, compressing, or bending deformation. He et al.^[^ ^43^ ^]^ have obtained a super‐stretchable LM foamed elastomer with coating the elastic composite of the silicon matrix into a porous LM structure, the resistance and thermal conductivity of which are increased following the tensile strain. Besides, Wang and coworkers^[^ ^44^ ^]^ have also achieved a similar soft LM/elastomer foam by covering LM on PDMS skeleton. Depending on the reservation of rich pore structure, the electrical/thermal property of LM and PDMS composite can be easily controlled by compressing extent. However, all these tunable electrical/thermal LMECFs without lasting external stimulation are unable to achieve stable deformation, which is limited by the intrinsic flexibility of elastomers.

As is well known, shape memory effect (SME) is the phenomenon that materials can be programed by external force and stimuli, keep various temporary shapes stably, and spontaneously recover to original shape under stimuli,^[^ ^45^, ^46^, ^47^ ^]^ the process of which is promising to solve the above mentioned problems. Currently, there are two strategies to endow LM composites with SME. One is employing LM and elastomer as macroscopical stationary phase and reversible phase,^[^ ^27^, ^28^, ^48^, ^49^ ^]^ reversibly, SME of which including shape memory reversible temperature and shape recovery/fix ratio is limited by the kinds, contents, and distribution of LM. The other way is the combination of LM and shape memory polymer (SMP), whose SME can be controlled by SMP with specific stimulus.^[^ ^38^, ^50^ ^]^ Accordingly, SME profits LM composites more superiorly in the fields of electrical and thermal conductive materials.

Herein, by the enhanced interaction between LM and SMP, LM shape memory polymer foam (LM‐SMF) is obtained, which displays the control to LM conductive path in non‐contact way. Interestingly, due to the tunable LM path, LM‐SMF possesses large‐scale regulation of resistance from electrical insulator to conductor, and control of thermal property with a thermal conductivity (*λ*) difference less than 0.021 W m^−1^·K^−1^, meaning that the sample can exhibit varieties of electrical/thermal properties during deformation by thermal stimulation. Basing on the excellent electrical and thermal dual‐regulation performance, LM‐SMF can be applied as a self‐feedback/‐warning electrical/thermal integrated switch or multifunctional infrared signal switch.

## 2 Results and Discussion

### 2.1 Macro/Micro Structure and Chemical Characterization of LM‐SMF

**Figure 1:**
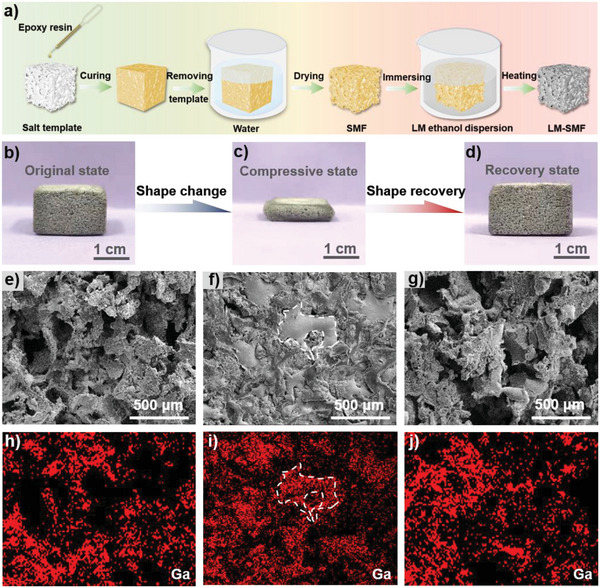
a) Schematic diagram of the preparation of LM‐SMF. b–d) Photographs and e–g) SEM images of LM‐SMF under original, compressive, and recovery states, respectively. h–j) Corresponding Ga element distributions by EDS. According to these results, the prepared LM‐SMF has excellent shape memory performance in both macro and micro scale.

The preparation process of LM‐SMF is shown in **Figure** **1**a. First, shape memory polymer foam (SMF) is fabricated by the salt template sacrifice method, which has a glass transition temperature (*T*_g_) ≈ 77 °C (Figure S1, Supporting Information) and good shape memory properties in both macro and micro structures (Figures S2 and S3, Supporting Information) under deformation temperature (≈100 °C). Then, by sonicating mixture of LM, ethanol, and (3‐Mercaptopropyl) triethoxysilane (MPTES), suspension with MPTES modified LM could be obtained (Figure S4, Supporting Information), which can be proved by the introducing of characteristic peaks of MPTES (Figure S5, Supporting Information)^[^ ^51^ ^]^ and formation of metal coordination bond between gallium and —SH group (Figure S6b, Supporting Information).^[^ ^52^ ^]^ Last, the SMF was immersed into prepared suspension, and the LM‐SMF could be obtained after simple heating process (removing solvents and completing the reaction). According to XPS spectra of SMF and LM‐SMF (Figure S7, Supporting Information), the characteristic elements (Ga, S, and Si) of LM and bridging agent MPTES can be detected on the skeleton of LM‐SMF. Besides, the LM‐SMF surface shows S2p peak at 160.6 eV and Si2p peak at 106.1 eV, corresponding to coordination bond between S—H bonds to the Ga ligand^[^ ^52^, ^53^ ^]^ and Si—O bond between Si—OH of MPTES and —OH of epoxy skeleton (Figure S8, Supporting Information).^[^ ^54^, ^55^ ^]^ Moreover, as shown in Figure S9, Supporting Information, the characteristic peak of LM‐SMF at 1000~1100 cm^−1^ was attributed to Si—O—C group, which illustrates the interaction of Si—OH and —OH group of epoxy resin.^[^ ^54^ ^]^ The results indicate that LM strongly loads on SMF skeleton by chemical interaction. Meanwhile, as shown in Figure 1b, different from the white SMF, the LM‐SMF displays gray color due to the existence of LM (containing Ga and Ga_2_O_3_, Figures S8 and S10, Supporting Information). Similar to pure SMF, rich pore structure still can be observed in LM‐SMF, and aggregating particles appear on foam skeleton (Figure 1e) at the same time, meaning that LM particles do not block the pores of SMF during loading process.

Meantime, through energy dispersive spectroscopy (EDS) images in Figure 1h, one can see that Ga element is distributed on the skeleton of foam uniformly, demonstrating the even loading of LM. Furthermore, heating LM‐SMF to temperature higher than *T*_g_ and pressing it under external force, the compressed LM‐SMF could be obtained after cooling to room temperature (Figure 1c). Through SEM image in Figure 1f, it is easily seen that the pores in original state are filled by continuous LM phase, which can also be certified by the enrichment of Ga element in corresponding EDS image. After reheating the compressed LM‐SMF, the height of foam returns to initial value. Excitingly, from SEM and corresponding EDS images (Figure 1g,j), it is clear that the stacking foam skeleton is also separated and LM is redistributed to the surface of skeleton. Those results prove that the prepared LM‐SMF has excellent shape memory performance, and the gathered bulk LM can redistribute following the shape recovery of SMF skeleton (certified by Figure S11, Supporting Information), which creates the possibility of electrical/thermal dual‐regulation in later work.

### 2.2 Electrical and Thermal Properties of LM‐SMF

**Figure 2:**
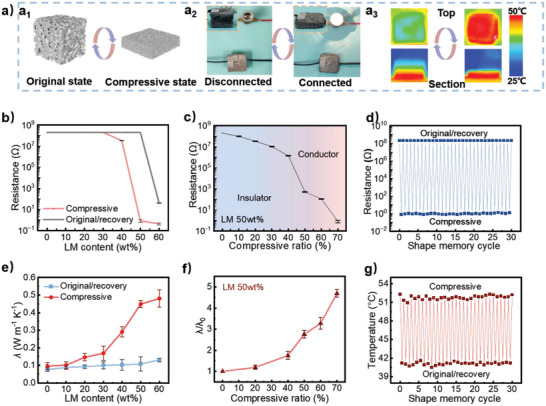
a_1_) Schematic diagram, a_2_) photographs of circuit status, a_3_) infrared thermometer images and insets (at top view and section view, a_3_) of original and compressed LM‐SMF. One can see that the LM‐SMF can reversibly show opposite electrical/thermal properties due to its great shape memory effect. b) Resistance changes of original/recovery and compressed LM‐SMF with different LM content. c) Statistic of resistance with different compressive ratio of LM‐SMF (50 wt% LM content), proving that the LM‐SMF can transform from electrical insulator to conductor by controlling compressive extent. d) Repeated transitions between electrical insulator (original/recovery state) and conductor (compressive state) can last at least for 30 times. e) The thermal conductivity changes of LM‐SMF with different LM content. f) The thermal conductivity changes following the increasing of compressive ratio. g) The stable surface temperature of LM‐SMF changes in 30 shape memory cycles. These results indicate that obtained LM‐SMF with 50 wt% LM has stably electrical/thermal controlling ability.

Furthermore, various electrical/thermal properties can be observed as demonstrated in **Figure** **2**a. When the original LM‐SMF (1.20 cm, as schematic in left of Figure 2a_1_) was connected to the circuit with bulb, the bulb could not be lit up (left in Figure 2a_2_), meaning that the sample was electrical insulator. However, when connected to the same circuit, the compressed sample (compressive ratio 70%, 0.36 cm height, as the schematic in right of Figure 2a_1_) lighted the bulb up, which showed different electrical conductivity (right in Figure 2a_2_), indicating that the LM‐SMF became a conductor under compressing. The ability to control the light on or off cannot be displayed by pure SMF (Figure S12, Supporting Information), demonstrating that LM plays a critical role in controlling various conductivity. Interestingly, in the similar process of shape change (compressed or recovered), the LM‐SMF could also display various thermal transmission. As shown in Figure 2a_3_, when the original foam was placed on heating stage at 80 °C, it was found that the stable surface temperature was 38 °C; while the compressed sample showed different stable surface temperature with 50 °C on the same heating stage, which rose significantly, indicating that the LM‐SMF in compressive state employed weaker thermal insulation than original state.

Therefore, to further investigate the effect of LM in electrical/thermal properties control, a series of samples with different LM contents were prepared. As shown in Figure S13, Supporting Information, following the increasing of LM content, more and more areas of SMF skeleton are coated by LM particles, and when the LM content is up to 60 wt%, the framework of SMF is almost totally covered by LM particles, which is set as the largest LM content. Then, to ensure great shape change performance, shape memory properties of series LM‐SMF are first studied (Figures S14 and S15, Supporting Information). Although the shape recovery rate (*R*_r_) decreases slightly, the foam with 60 wt% LM content still has a relatively high *R*_r_ with 98.2%. Meantime, the shape recovery time (at 100 °C) of LM‐SMF decreases from 140.5 to 81.7 s as the LM content increases, indicating that the content of LM is benefit to improve the *R*_r_.

To further investigate the effect of LM content, the resistance of LM‐SMF under different states is measured (Figure 2b). When the LM content is less than 30 wt%, the resistance of samples at both original and totally compressive state keeps at 200 MΩ. However, when LM content increases to 60 wt%, all the foam variations display much lower electrical conductivity with a resistance smaller than 41 Ω (Figure S16, Supporting Information). As LM content is 50 wt%, sample at original and compressive states displays electrical conductor and insulator property, respectively, and the largest resistance difference between two variations can be realized. Similarly, the *λ* of obtained foam at original and compressive state is tested by laser thermal conductivity meter. As exhibited in Figure 2e, higher LM content and compressing procedure can result in higher thermal conductivity. In detail, with the increase of LM content, *λ* of LM‐SMF increases from 0.079 to 0.134 W m^−1^ K^−1^, and *λ* difference between two states also increases gradually. As LM content is as high as 60 wt%, the *λ* difference reaches to ≈0.349 W m^−1^ K^−1^. Hence, according to the above results, especially, 50 wt% is selected for the optimum LM content of obtained LM‐SMF, which allows the formation of conductive path and wide‐range regulation in this work.

By the method shown in Figure S17, Supporting Information, LM‐SMF with various thickness can be prepared (Figure S18, Supporting Information). Following the increase of the compressive ratio, the foam skeleton stacking is closer, and LM fills the pores of foam gradually (Figure S19, Supporting Information). As a result, as shown in Figure 2c, when the compressive ratio of the LM‐SMF changes from 0% to 70%, LM‐SMF converts from the insulator (200 MΩ) to conductor (0.8 Ω), demonstrating that dual‐regulation of conductivity can be achieved by controlling compression extent. More interestingly, through same operation, regulation of thermal property can be obtained. Increasing of compressive ratio to 70%, the *λ* of sample increases to ≈0.509 W m^−1^ K^−1^, which is 4.71 times of the initial value (0.108 W m^−1^ K^−1^) as shown in Figure 2f. Meantime, various *λ* endows different stable surface temperatures when the samples are put on heating plate with same temperature (Figure S20, Supporting Information), meaning that the thermal property, including *λ* and stable surface temperature, can be fine‐tuned by a simple compressing process with a control accuracy ≈0.021 W m^−1^ K^−1^ and ≈3–5 °C. Such electrical/thermal integrated dual‐regulation materials are rarely reported in previous works. Furthermore, contributing to excellent cyclic compression of LM‐SMF from great shape memory effect (Figure S21, Supporting Information) and stable LM distribution after 30 cycles (Figure S22, Supporting Information), the regulation of electrical and thermal properties of the sample also has excellent cycle stability (Figure 2d,g), which can employ at least 30 cycles of reversible transitions of electrical conductor–insulator and surface temperature change. The great cycle stability builds basics for the practical applications of LM‐SMF.

### 2.3 The Mechanism of Electrical/Thermal Coordinated Dual‐Regulation of LM‐SMF

**Figure 3:**
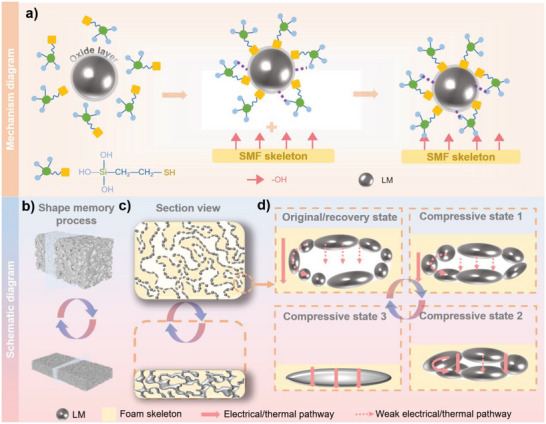
a) Bridging mechanism of LM particles and SMF skeleton, indicating that LM particles can tightly “grow” on the surface of SMF skeleton through MPTES. b) Schematic illustrations of shape memory process. c) Section view of original/recovery (top) and compressed (bottom) LM‐SMF. d) The electrical and thermal pathway state follows the change of pressing extent. One can see that the reversible shape changes lead to various electrically/thermally conductive pathway, realizing the dual‐regulation of electrical resistance and thermal conductivity.

To investigate the mechanism of the great electrical/thermal coordinated dual‐regulation of LM‐SMF, electrically and thermally conductive mechanism of LM‐SMF has been clarified at first. In this work, epoxy resin shows electrical insulation and LM is the only path for electric current. Meanwhile, thermal conduction is the main way of thermal transmission, the media of which contains air (0.023 W m^−1^ K^−1^),^[^ ^56^ ^]^ foam skeleton based on epoxy resin (0.077 W m^−1^ K^−1^, Figure 2e), and LM (33.49 W m^−1^ K^−1^).^[^ ^57^ ^]^ Therefore, controlling connecting status of the LM is crucial. However, due to the high surface tension of LM, it is not easy for bulk LM to enter porous foam due to capillary of resistance,^[^ ^23^, ^58^ ^]^ neither LM contacting control. To solve this problem, by mixing and sonicating MPTES, LM, and ethanol, uniformly dispersed LM suspension with LM particle size of 5–20 µm (Figure S4, Supporting Information) can be prepared, which can make the process of forming LM‐SMF composite more easily. In detail, as shown in Figure S23, Supporting Information, the possible existing hydrogen bonds (between Si—OH groups of MPTES and oxide layer [Ga_2_O_3_] of LM) and metal coordination bonds (between —SH groups of MPTES and gallium) make MPTES stably combine with LM particles.^[^ ^59^, ^60^, ^61^ ^]^ Besides, the formation of Si—O—C group (Figure S9, Supporting Information) reinforces interaction between MPTES and polymer matrix. Therefore, MPTES acts as a “bridge” between LM and SMF skeleton, enormously improving the bonding force between LM particles and SMF skeleton and ensuring the stability of deformation process (**Figure** **3**a).

Hence, in permanent shape state (top of Figure 3b), as illustrated in Figure 3c (top), the continuous structure only contains air and epoxy resin foam skeleton, the conformation of whose molecular chains has higher entropy (Figure S24, Supporting Information) and resides in a thermodynamically stable state. However, the LM just distributes on the foam skeleton as discontinuous particles and the oxide surfaces of LM particles are electrically insulated,^[^ ^41^ ^]^ resulting in no electrical or thermal transfer path being formed. Namely, the LM‐SMF displays a great electrical insulation (200 MΩ) and very low *λ* (0.108 W m^−1^ K^−1^). When the foam is heated to the temperature higher than *T*_g_, it becomes soft due to the obvious decrease of storage modulus (Figures S1 and S25, Supporting Information) and the molecular chain is activated. In this situation, the foam can be pressed and become thinner under external force. Keeping the external force and cooling the foam to temperature lower than *T*_g_, the molecular chains are frozen in a lower entropy state, and LM‐SMF in temporary compression shape can be obtained.^[^ ^47^ ^]^

As shown in Figure 3d, following the increasing of stacking extent of foam skeleton, the discontinuous LM particles gradually contact to each other, and the oxide surfaces are broken by squeezing and gathering to bulk LM, forming continuous phase finally (bottom in Figure 3c). During that process, the electrical/thermal conductive pathway changes from epoxy foam skeleton to LM pathway, largely improving the electrical and thermal conductivity of LM‐SMF. Correspondingly, the resistance of sample is also gradually decreased, realizing the transition from electrical insulator (200 MΩ) to conductor (0.8 Ω). Interestingly, the above mentioned situation only happens as LM content is 50 wt%. LM cannot form continuous pathway at any compressive ratio when the LM content is lower. When the LM content is much higher (up to 60 wt%), a large control range cannot be obtained, and there is exudation of LM bulk when LM content reaches 60 wt% during the first deformation cycle, which is unfavorable for the following applications. Moreover, during the deformation process, the foam maintains great chemical stability (Figure S26, Supporting Information), ensuring electrically/thermally controllable stability transition.

### 2.4 Self‐Feedback/‐Warning Integrated Smart Switch

**Figure 4:**
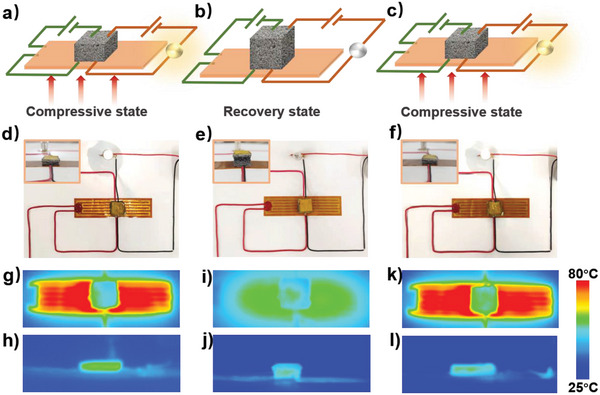
a–c) Schematic diagrams of circuits with self‐feedback/‐warning integrated smart switch on compressive state (connected), recovered state (disconnected), and re‐compressive state (reconnected). When LM‐SMF is compressed, the circuit is in normal working state, and when the bottom heating film is over‐heated, LM‐SMF will cause the shape recovery, leading to phenomenon of circuit self‐protection. d–f) Pictures of smart switch on normal (compressive), self‐warning (spontaneous recovery), and rework (compressive) state, corresponding to states in (a,b,c), respectively. g–l) IR images of samples in top and section view. g,h) Demonstrates that when the heating film is ≈75 °C ± 3 °C, the circuit is in normal working state. i,j) Displays low temperature of heating film due to broken circuit after overheating process. k,l) Renormal working state following the re‐pressing process of LM‐SMF.

Obtained LM‐SMF can be potentially used as a designable self‐feedback/‐warning integrated smart switch to warn or adopt measures for dangerous thermal signal relying on its capability of quantitative electrical/thermal control. **Figure** **4** illustrates the application in self‐feedback/‐warning heating system. As shown in Figure 4a, to display the self‐feedback/‐warning function, LM‐SMF is connected to two independent circuits (including heating and warning circuits) at the same time. When LM‐SMF with 70% compressive ratio is utilized (inset photograph in Figure 4d), both circuits can be connected because of its great electrical conductivity (0.8 Ω). As revealed in Figure 4g,h, the surface temperature of heating film and LM‐SMF is ≈75 °C ± 3 °C and 50 °C, respectively, which is marked as safe temperature in this work (Figure S27, Supporting Information). Meantime, the indicator light in warning circuits keeps bright, demonstrating the normal operation of this system.

However, the heating system sometimes will be overheated (160 °C, Figure S28, Supporting Information), which is urgently needed to give a warning signal and be disconnected to avoid the danger. In this situation, the shape recovery of LM‐SMF will be triggered at high heating temperature. As mentioned above, following the shape recovery, the resistance of LM‐SMF gradually increases until it becomes an electrical insulator. As a result, as the resistance of LM‐SMF in heating circuit becomes higher and higher, the power for heating film will be lower and lower (Figure 4i,j), meaning that the overloading danger will be removed by automatic action of LM‐SMF. Meantime, the indicator light is also extinguished during the shape recovery process (Figure 4b,e), warning that the heating system is in normal state. It makes more sense that thermal conductivity of LM‐SMF is low at uncompressed state, ensuring a relatively stable surface temperature of LM‐SMF.

More interestingly, if the problem of heating system is solved in time, the recovered foam might be repressed immediately while there is enough residual heat, meaning that the heating and warning circuits can be restarted in situ (Figure 4c,f,k,l). Undoubtedly, if the open state of circuit is kept for long time, the foam can still be taken and reshaped separately, which can also reboot the heating system to the normal working station. In another word, this smart switch not only displays self‐feedback/‐warning function but also can be reversibly used for many times on the basis of its excellent shape memory effect.

In the future, larger area LM‐SMF is possible to be obtained to work as thermostatic heater with self‐feedback/‐warning functions. Instead of placing on general heating film directly, LM‐SMF can efficiently prevent overheating damage caused by short‐circuit thermal runaway. Besides, because the resistance and thermal conductivity of LM‐SMF can be controlled by adjusting its compressive ratio, various stable heating temperature on single heating systems can be realized.

### 2.5 Tunable Infrared Signal Camouflage Switch

**Figure 5:**
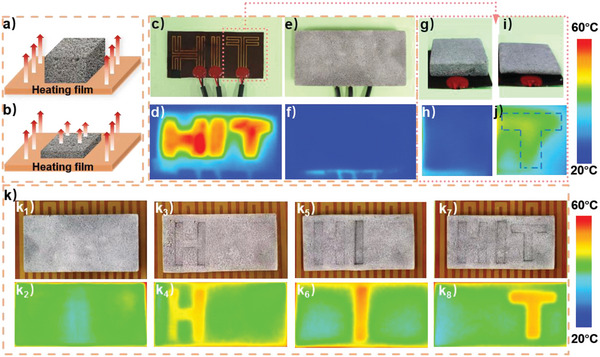
a,b) Different thermal transmission states of original/recovery and compressed LM‐SMF, showing that infrared signal can be hidden or displayed by controlling the thickness of SMF. c) Picture of heating film with the pattern “HIT.” d) IR image of the pattern “HIT” on heating film without anything coating. e) Picture of original LM‐SMF on the patterned heating film. f) IR image of (e), proving that the IR signal can be successfully hidden by the LM‐SMF. g,i) Pictures of uncompressed and compressed LM‐SMF on local heating film with pattern “T”. h,j) Corresponding IR image of (g,i), demonstrating that the prepared LM‐SMF can switch on or off the IR signal transfer. k) Reversible counterfeiting of IR signals. When putting original LM‐SMF on a generous heating film (k_1_), no IR pattern can be seen (k_2_). In addition, when the LM‐SMF is compressed with the pattern “H,” “I,” and “T” in turn (k_3_, k_5_, and k_7_), the corresponding forge IR signal can be observed (k_4_, k_6_, and k_8_).

In addition, based on excellent thermal conduction control and shape memory performance, LM‐SMF obtained in this work can also realize tunable infrared stealth function, which can be widely used in the fields of military, aerospace, wearable devices and so on, and can satisfy complex environments. As displayed in **Figure** **5**a, putting the original LM‐SMF on a common heating film (heating temperature 70 °C), the good thermal insulation property can prevent thermal transmission, meaning that no thermal signal can be detected due to existence of LM‐SMF. However, compressing LM‐SMF can connect part of the thermal path, as a result of whose infrared signal is easy to be detected (Figure 5b). Based on this feature, a series of infrared stealth materials can be designed. To exhibit special infrared information, the heating wire is arranged to the shape of “HIT” (Figure 5c). When there is no LM‐SMF on heating film, the clear infrared signal of “HIT” can be observed (Figure 5d), meaning that the thermal signal of “HIT” is exposed in infrared detection. However, when the same film is covered by LM‐SMF in the uncompressed state, the thermal transmission is greatly attenuated by the foam and the “HIT” pattern disappears (Figure 5e,f), indicating that LM‐SMF has great ability of infrared signal camouflage.

In fact, sometimes the infrared information is required to selectively and reversibly appear or disappear. Taking signal “T” as an example (Figure 5g–j), placing uncompressed and compressed foam on heating film in working state, the infrared signal “T” can be hidden and displayed, respectively. Besides, our sample can also be used as infrared signal jamming device, interfering with infrared monitoring. Namely, by special design, nonexistent infrared signal can be created. As shown in Figure 5k_1,_k_2_, no thermal signal can be detected by coating original LM‐SMF on a general heating film (surface temperature ≈70 °C). However, when creating a pattern “H” on the foam (Figure 5k_3_), the infrared signal “H” can be monitored by infrared thermal imager (Figure 5k_4_) due to inhomogeneous thermal transmission.

More interestingly, based on good shape memory performance, the pressing pattern can be erased and rewritten by a simple heating and repressing process. Figure 5k_5__,_ k_7_ displays the pattern “I” and “T” sequentially, and both corresponding infrared signals can be detected in Figure 5k_6_,5k_8_. Even though the weak press traces are residual (such as “H” and “I” in Figure 5k_7_), the infrared signals are not be affected due to enough shape recovery of pressing part. In addition, if all patterns are erased, no infrared can be monitored again (Figure S29, Supporting Information). Therefore, the obtained LM‐SMF can be reversibly utilized as pseudo infrared target to confuse the external observation. What's more, the sample is also potentially applied in heating system with real‐time monitoring function, electrothermal sensor, and so on.

## 3 Conclusion

In this work, we demonstrate a porous shape memory composite foam with electrical/thermal coordinated dual‐regulation, which displays tunable and reversible resistance (between 200 MΩ and 0.8 Ω) and *λ* (between 0.108 W m^−1^ K^−1^ and 0.509 W m^−1^ K^−1^). Contributing to the great thermal response shape memory performance of SMF, it is possible to get various continuity of LM, which is the electrically/thermally conductive pathway in LM‐SMF mainly. Therefore, by controlling the compressive ratio, LM‐SMF can exhibit excellent electrical and thermal dual‐regulation performance. Furthermore, in view of the special electrical/thermal coordinated dual‐regulation, the novel applications of LM‐SMF as a smart switch for self‐feedback/‐warning circuits and infrared signal camouflage are also reported. This work advances an idea to achieve stable electrical/thermal integrated control materials and first realize it based on shape memory foam and LM. Given this great electrical/thermal regulation ability, LM‐SMF can also be widely used as integrated material in thermal controllable insulation building materials, or electronic equipment requiring thermal control. Meantime, this concept can easily be broadened to other SMPs with non‐contact external stimulus, such as electric, light, or magnetic.

## 4 Experimental Section

### Materials

LM (gallium 100%, melting point 29.8 °C) was obtained from Shenyang Jiabei Trading Co., Ltd. (Shenyang, China). E‐44 epoxy resin (E‐44) was purchased from the China Petrochemical Corporation. Polyetheramine (D‐230) and MPTES were purchased from Shanghai Macklin Biochemical Co., Ltd. Sodium chloride and ethanol were provided from Shanghai Aladdin Bio‐Chem Technology Co., Ltd. All the above materials were used without any treatment.

### Fabrication of SMF and LM‐SMF

First, 10 g E‐44 and 3.4 g D‐230 were mixed and infiltrated into the prepared salt template by vacuum impregnating. Then, the mixture and salt template were cured at 80 °C for 2 h and at 130 °C for 2 h. SMF could be obtained after the procedure of removing the salt template (washing by deionized water and drying). The thiol‐modified LM ethanol suspension was fabricated by sonicating a mixture of 2 g LM, 50 mL ethanol, and 20 µL MPTES for 30 min. Last, SMF was immerged into thiol‐modified LM ethanol and then dried at 60 °C for 4 h, and LM‐SMF with different LM content could be achieved after repeating the above process for different times. LM‐SMF was prepared in the size of 20 × 20 × 12 mm. If there is no obvious description, the results in this article are the sample test results of LM‐SMF with 50 wt% LM content.

### Characterization of SMF and LM‐SMF

The micromorphology of LM‐SMF was observed by field emission scanning electron microscopy (SEM), (SUPRA 55, Carl Zeiss, Germany). Rigaku Ultima IVXRD (Japan) was used to test X‐ray diffraction (XRD) of SMF and LM‐SMF, whose scanning speed was 5° min^−1^. X‐ray photoelectron spectroscopy (XPS) was analyzed by Thermo Fisher Nexsa (America). *T*_g_ was obtained by TA Q800 (America) dynamic thermomechanical analyzer (DMA). Mechanical properties were measured by INSTRON 5969 universal testing machine (America).

### Shape Memory Performance Test

The shape memory performance test process is shown in Figure S30a, Supporting Information. The initial height of sample is *h*_0_. After heating the sample to 100 °C for 10 min and deforming the sample by compressing with external force, the height of sample becomes *h*_i_. Then, the temporary shape of sample is fixed and the compressed sample height is *h*_f_ by cooling the sample to room temperature for 10 min with the sustained external force. The sample recovers to initial shape and the height becomes *h*_r_ by heating the compressed sample to 100 °C again for 10 min. Compressive ratio of sample is marked as *R*_c_, and shape recovery rate is marked as *R*_r_. The calculation formula is shown below.

(1) $$R_{c}=h_{f}/h_{0}\times 100\%$$

(2) $$R_{r}=h_{r}/h_{0}\times 100\%$$

### Characterization of Electrical and Thermal Properties

The resistance and surface temperature measurements of the sample are shown in Figure S30b,c, Supporting Information, and multimeter (UNI‐T, UT39A+, China) is used to measure the resistance between bottom and up side (the shortest distance along the pressing direction), which is always the average value for at least six testing positions. Laser thermal conductivity meter LFA 467 (America) determined the thermal conductivity. The stable surface temperature of samples (average of six testing points) and IR images were obtained by Testo 865 thermal imager. The samples were heated by PI heating film loaded with DC current with heating voltage ranging from 3 to 12 V.

## Conflict of Interest

The authors declare no conflict of interest.

## Supporting information

Supporting Information

## Data Availability

Research data are not shared.
