# Supplementary material for: Designable Electrical/Thermal Coordinated Dual‐Regulation Based on Liquid Metal Shape Memory Polymer Foam for Smart Switch
Source: Adv Sci (Weinh). 2023 Jan 19;10(8):2205428. doi: 10.1002/advs.202205428 (PMC10015848; doi:10.1002/advs.202205428)
Supplement: Supplementary file 1 — Supporting Information [file ADVS-10-2205428-s001.pdf]

Supporting Information

Designable Electrical/Thermal Coordinated Multi-Regulation Based on Liquid Metal Shape Memory Polymer Foam for Smart Switch

Ruoxi Zhao[1], Sibo Kang[2], Chao Wu[1], Zhongjun Cheng[1], Zhimin Xie[3], Yuyan Liu[1*], Dongjie Zhang[1*]

[1]*School of Chemistry and Chemical Engineering, Harbin Institute of Technology, Harbin 150001, P. R. China*

[2]*State Key Laboratory of Marine Coating, Marine Chemical Research Institute Co., Ltd., Qingdao 266071, P. R. China*

[3]*National Key Laboratory of Science and Technology on Advanced Composites in Special Environments, Harbin Institute of Technology, Harbin 150080, P. R. China*

***Corresponding authors:**

Dongjie Zhang (djzhang@hit.edu.cn)

Yuyan Liu (liuyy@hit.edu.cn)

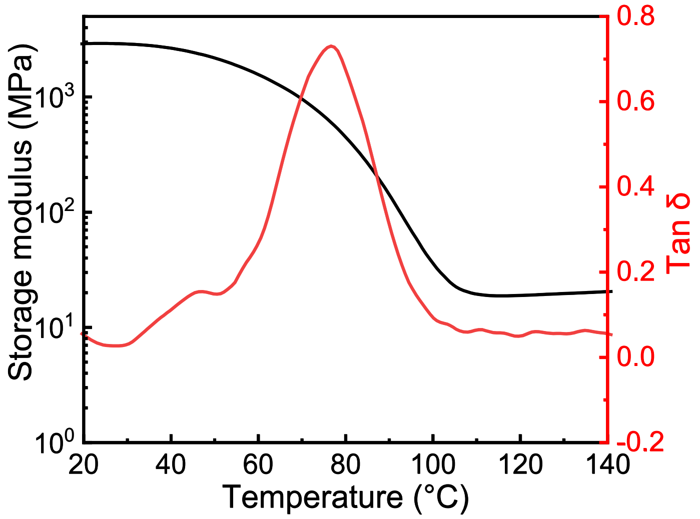

Figure S1. DMA curves of shape memory resin sample. Glass transition temperature (T_g) is about 77 °C, determined by the position of Tan δ peak. The storage modulus reaches to 2.94×10^3 MPa at 20 ℃ and decreases to 11 MPa at 100 ℃. The huge difference in storage modulus indicates the possibility of shape memory performance of shape memory resin, which also contributes to the shape memory ability of shape memory foam (SMF).

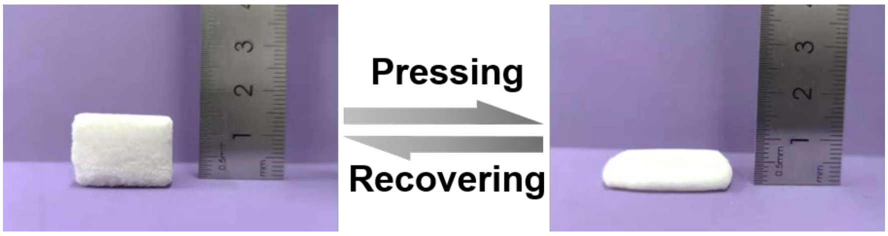

Figure S2. Pictures of original/recovery and compressed SMF, showing the excellent shape memory effect of SMF.

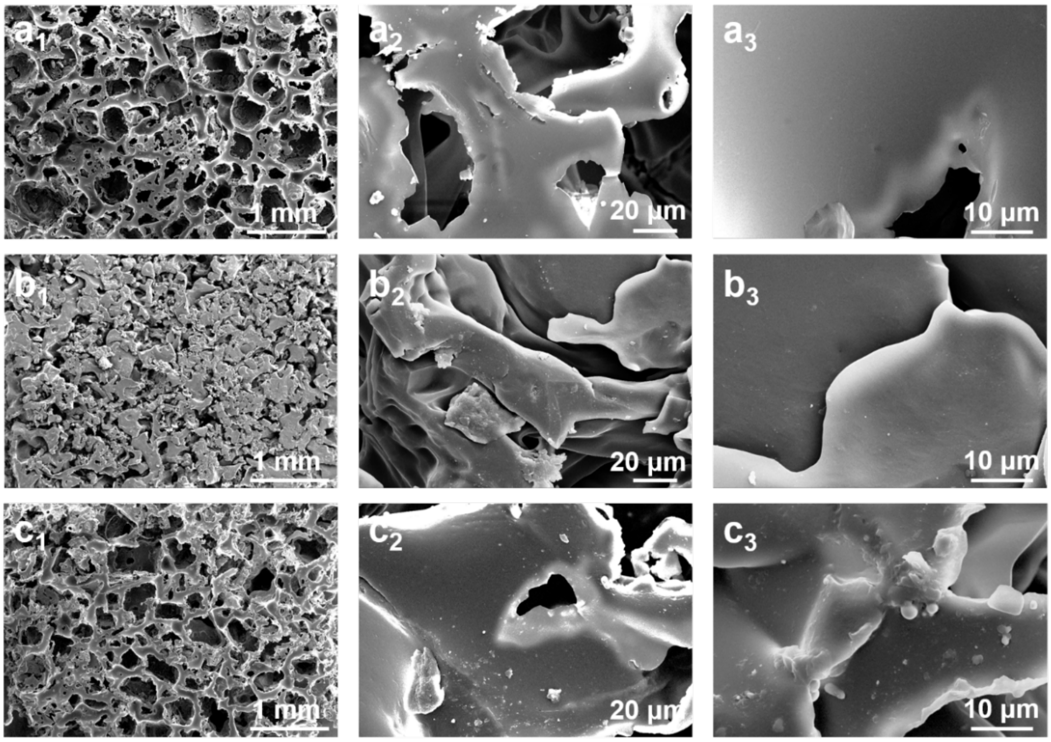

Figure S3. SEM images of SMF in different state and magnification. a_1)-a_3) Original SMF, b_1)-b_3) compressed SMF, and c_1)-c_3) recovered SMF. As shown in SEM images, original SMF (a_1), (a_2) has porous structure with the pore size of 100 ~ 500 μm, and the surface of SMF skeleton is smooth (a_3). After compressing, the large pore of SMF becomes smaller pore and the skeleton is stacked together, but the skeleton surface is still smooth (b_1)- (b_3). After recovering, the pore structure and skeleton surface are the same as the original state (c_1)- (c_3), which indicates that SMF has great shape memory ability at the micro scale.

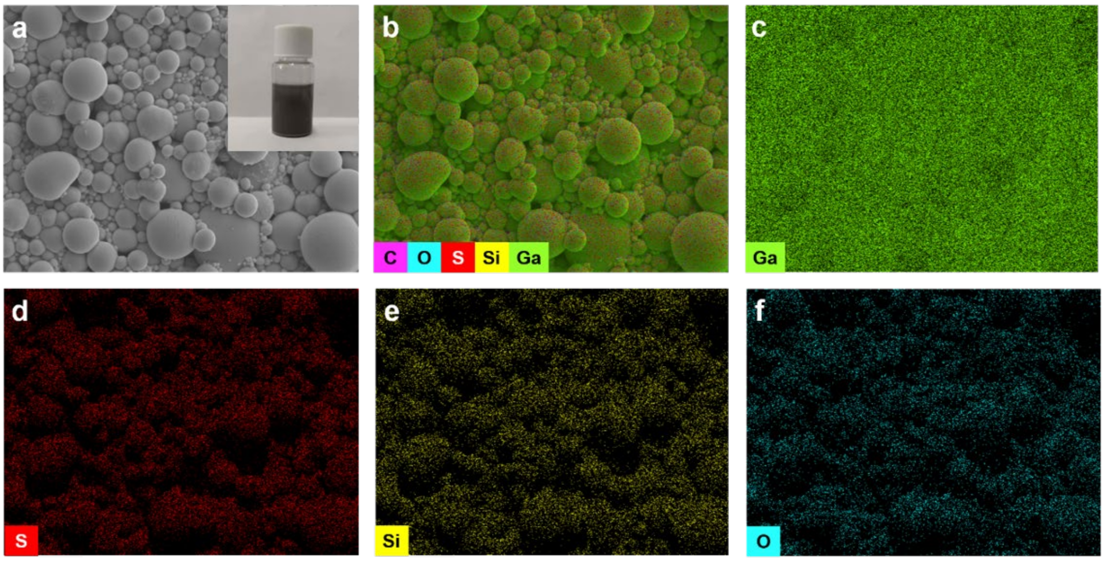

Figure S4. SEM and EDS images of thiol-modified liquid metal (LM) ethanol dispersion. a) SEM image and insert of MPTES modified LM particles (m-LMP). b) - f) EDS images of m-LMP. SEM image of m-LMP shows that the particles are in size of 5 ~ 20 μm and there are no particle aggregation occurring. The characteristic element S, Si, and O in EDS images indicate that LM particle is evenly modified by MPTES.

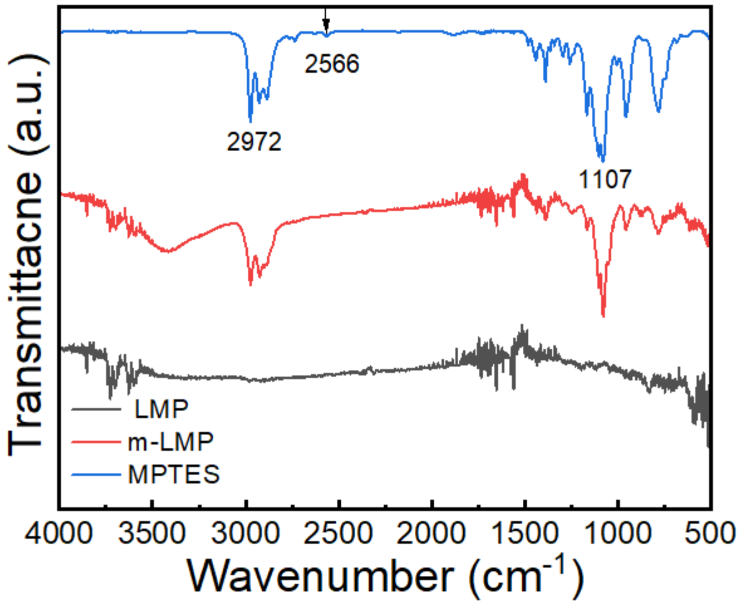

Figure S5. FT-IR spectrum of MPTES, unmodified LM particles (LMP) and m-LMP. It can be seen that there is no characteristic peak in LMP obviously. By the modification of MPTES, the peaks of methylene, Si-O, and -OH group appear at 2972 cm^{-1}, 1107 cm^{-1}, and 3400 cm^{-1} in m-LMP, respectively, which belong to the characteristic peak of MPTES. Meantime, the peak of thiol-terminal group (at 2566 cm^{-1}) disappears in m-LMP, meaning the formation of metal coordination bond between LM and -SH group.[1-3] The above results indicate that MPTES has been introduced to LMP.

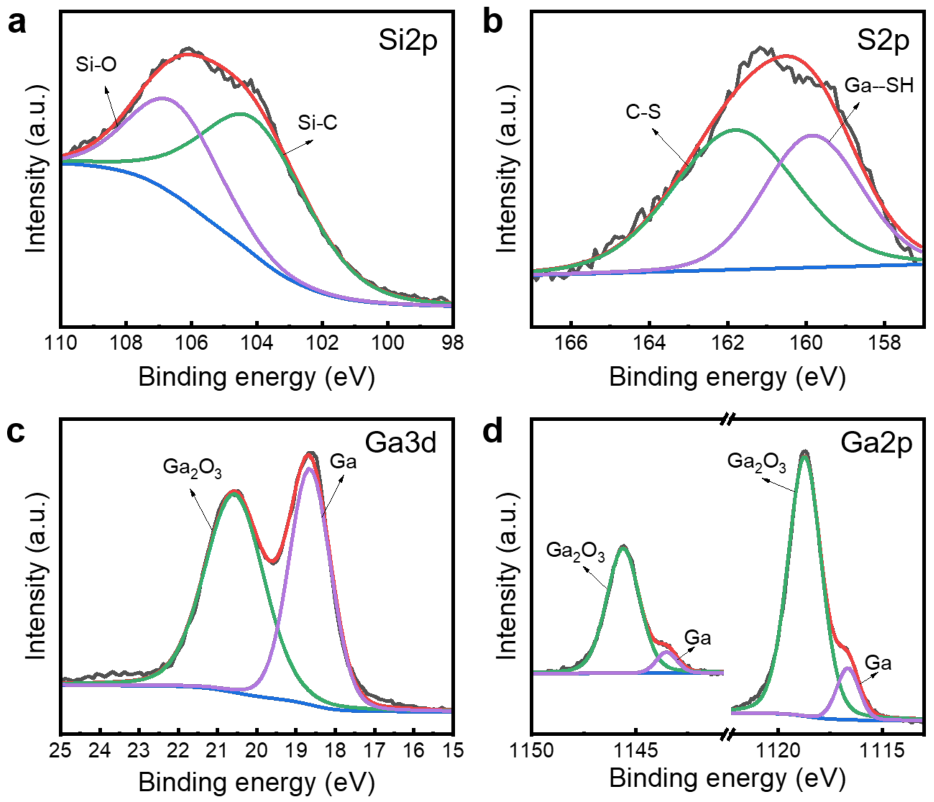

Figure S6. XPS high-resolution spectrum a) Si2p, b) S2p, c) Ga3d and d) Ga2p of m-LMP. The peaks located at 106.5, 104.0, and 161.4 eV are ascribed to the bonds of Si-O, Si-C, and C-S of MPTES, respectively. The peak at 159.4 eV of S element indicates the formation of metal coordination bond between Ga and -SH.[2,4] The high-resolution spectrum of Ga3d and Ga2p confirm the existences of Ga_2O_3 and gallium in m-LMP. These results further indicate the successful modification of LM.

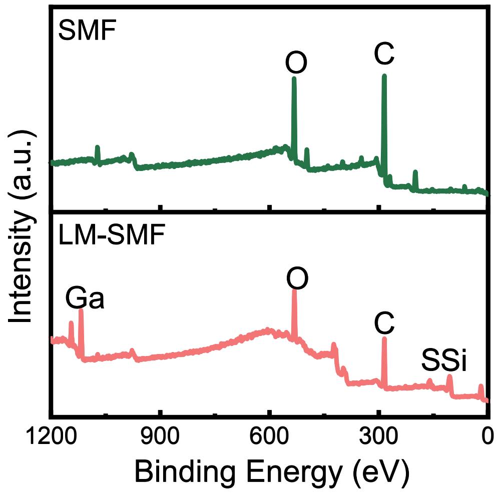

Figure S7. XPS spectra of SMF and LM shape memory foam (LM-SMF). As a result, Ga (1118 eV), S (160 eV) and Si (105 eV) elements appears in LM-SMF after compositing LM particles, which preliminarily illustrates that LM particles and MPTES combine with SMF.

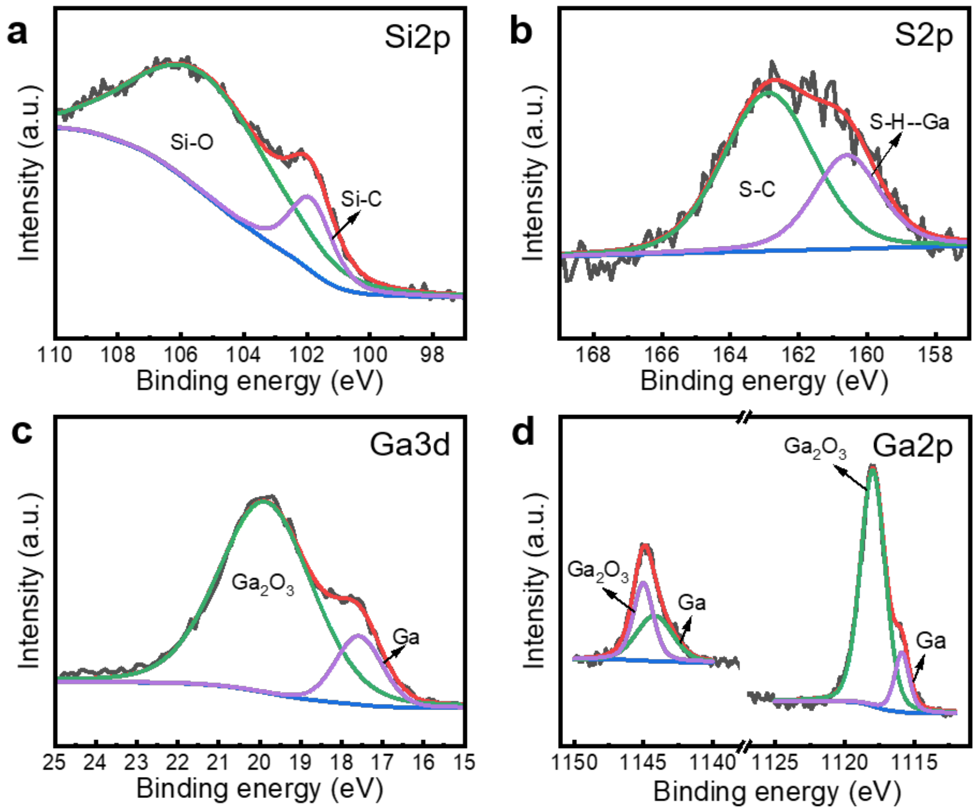

Figure S8. XPS spectra of different elements of LM-SMF. a) Si2p, b) S2p, c) Ga3d and d) Ga2p. The spectrum of S element shows that the peak of 160.6 eV is S-H and Ga ligand binds.[1-4] Besides, binding energy of 106.08 eV corresponds to Si-O bond,[5,6] which demonstrates the reaction between Si-OH of MPTES and -OH in epoxy skeleton. The binding energy of 17.6 eV (Ga3d), 1143.6 eV and 1115.3 eV (Ga2p) belong to Ga element (Figure d). The binding energy of 20.0 eV (Ga3d), 1144.5 eV and 1117.5 eV (Ga2p) indicate Ga_2O_3 of LM particles surface, respectively.[2,3]

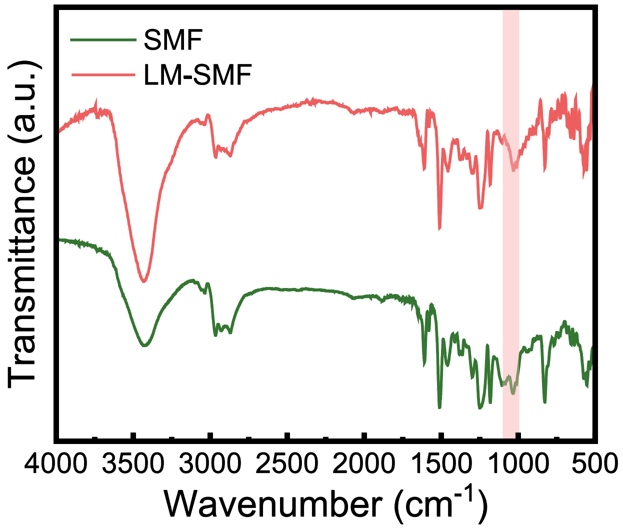

Figure S9. FT-IR spectrum of SMF and LM-SMF. By comparison, the characteristic peak at 1000 ~ 1100 cm^{-1} LM-SMF is attributed to Si-O-C group, which illustrates the reaction between Si-OH of MPTES and -OH group of epoxy resin.[5,6] As a result, by the analysis of XPS and FT-IR, one can see that LM stably "grow" on the surface of SMF skeleton.

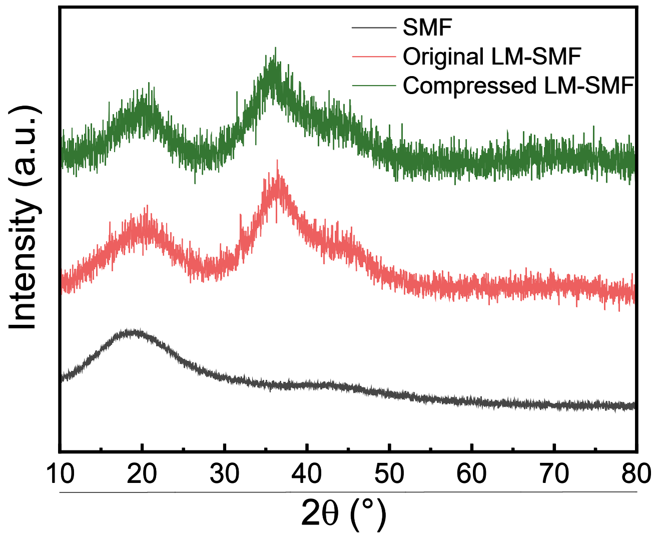

Figure S10. XRD spectrum of SMF, original LM-SMF, and compressed LM-SMF. The wide diffraction peak at 20 degree belongs to SMF. The peaks at 35 and 45 degree can be attributed to α-Ga_2O_3 and γ-Ga_2O_3 of the oxide layer, respectively.[7] The wide diffraction peak indicates the low crystallinity of Ga_2O_3. Meantime, it can also be seen that the compressive process does no change with the chemical structure of Ga element, meaning that the chemical structure of LM-SMF is stable during the shape change process.

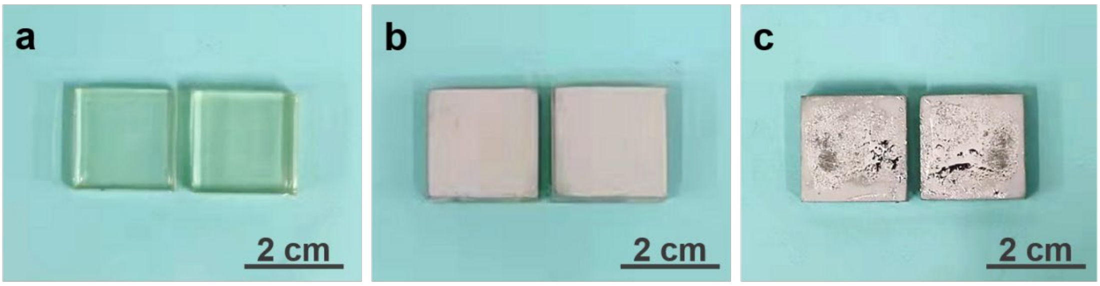

Figure S11. Macro demonstration pictures in separation process of LM-SMF skeleton. a) Pure shape memory resin sample. b) Shape memory resin sample with LM particles. c) Shape memory resin sample with LM particles after the process of squeezing and separating, and LM adhered on the shape memory resin sample well. To prove LM can adhere on the SMF skeleton in the compressive process well, the shape memory resin sample was prepared to simulate the contact state between SMF skeletons during extrusion and separation. The sides of shape memory resin sample with LM particles were squeezed and separated, and the surface changes from rough to metallic luster obviously. Parts of bulk LM connected together due to the broken of LM particles causing by the surface state change. What's more, LM adheres on the surface evenly with no aggregation. Because of the binding force between LM and skeleton,[8] LM won't be separated from the skeleton, and still adheres on the skeleton during the process of extrusion and separation.

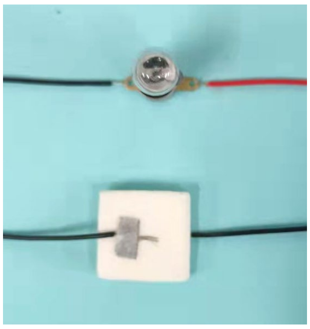

Figure S12. Photograph of compressive SMF (70% compressive ratio) connecting in the circuit with bulb (3V), proving that compressive SMF cannot light the bulb in the circuit (3V).

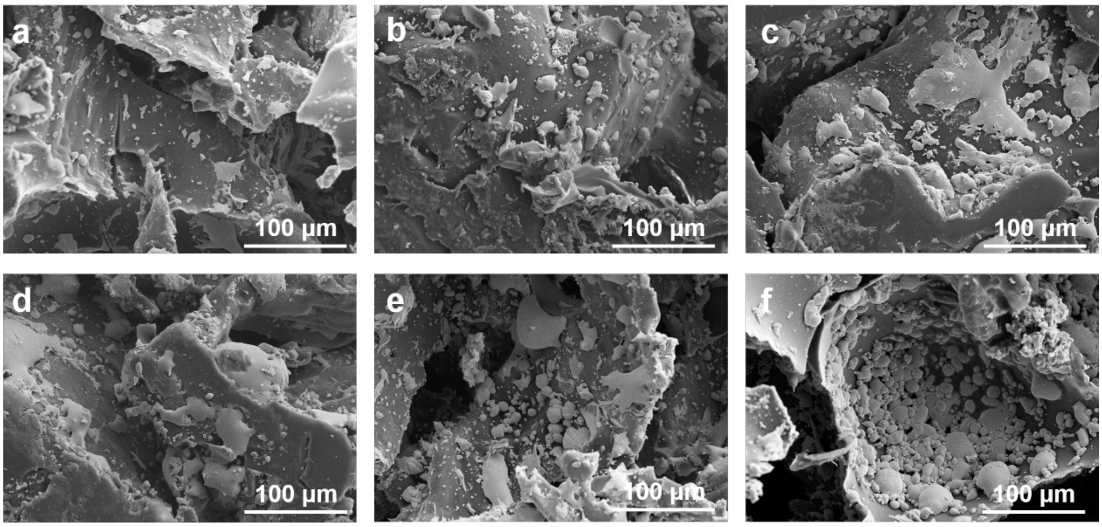

Figure S13. SEM images of LM-SMF with different LM content. a) 10wt%, b) 20wt%, c) 30wt%, d) 40wt%, e) 50wt% and f) 60wt%. SEM images display that more and more LM particles adhere on the skeleton of foam with the increase of LM content.

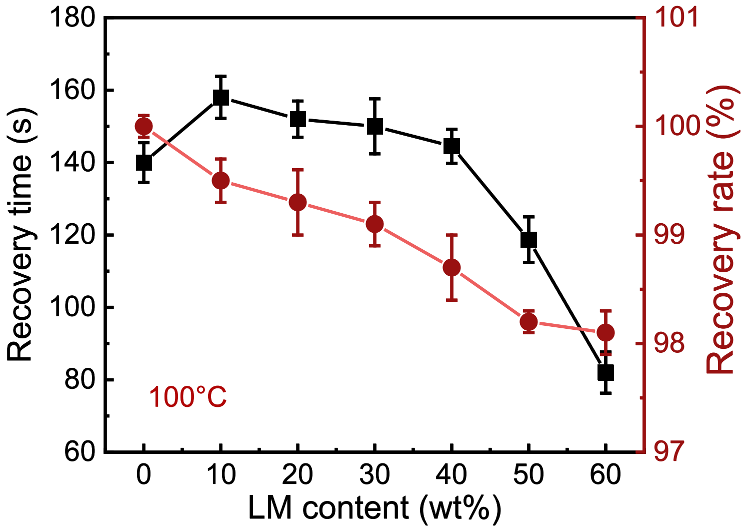

Figure S14. Shape recovery time and recovery rate of deformed LM-SMF with various LM content at 100 °C.

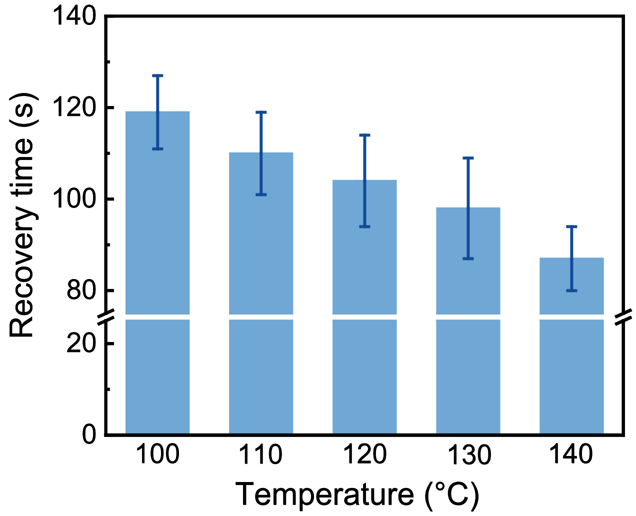

Figure S15. Shape recovery time change with recovery temperature of LM-SMF (50wt% LM content). Herein, the shape recovery process was happened in an oven, which is benefit for evenly heating. As a result, the shape recovery time is reduced from 119.2 s (100 °C) to 87.1 s (140 °C) with the increase of shape recovery temperature. The increase of temperature will accelerate the thermal motion of molecular chain in matrix resin, so that the shape of molecular chain can be restored to initial state faster, and the

shape memory recovery time of LM-SMF can be reduced.

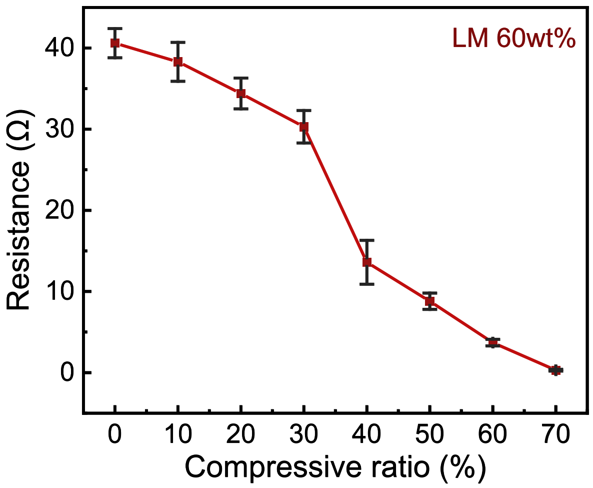

Figure S16. The resistance of LM-SMF (with 60wt% LM content) changes with compressive ratio. The resistance of LM-SMF in 60wt% LM content is only 40.4 Ω and LM-SMF is conductor in the initial shape (0% compressive ratio). After compressing gradually, the resistance reduces correspondingly and becomes 0.3 Ω in 70% compressive ratio. However, the resistance is only two orders of magnitude during shape changing process.

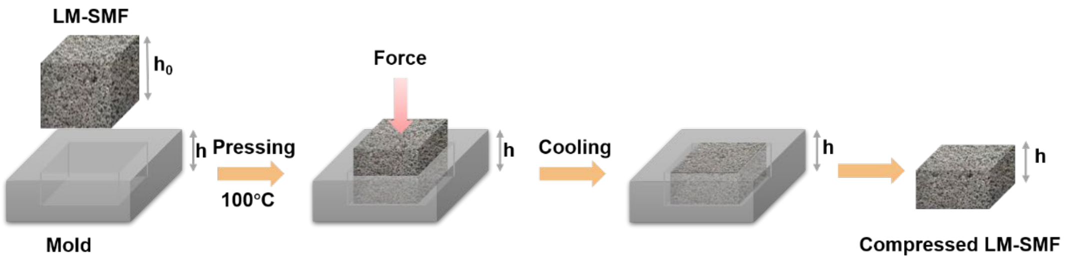

Figure S17. Schematic diagram of shaping LM-SMF with mold to different compressive ratio. In this work, LM-SMF with different compressive ratio is obtained by shaping in height-adjustable mold. Firstly, LM-SMF (height h_0) is put in the mold (height h). Then the foam is heated to 100 °C and compressed with sustained external force until the height is equal to h. The height of LM-SMF change from h_0 to h after a simple hot pressing and cooling process. Here, the compressive ratio is defined as $h/h_0 \times 100\%$.

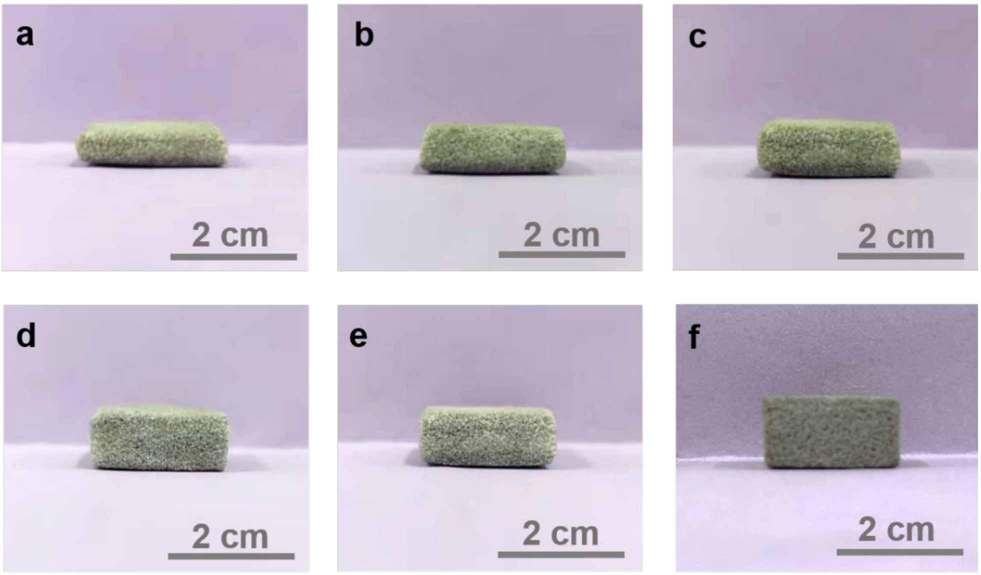

Figure S18. Photographs of LM-SMF (50wt% LM content) with various compressive ratio. Compressive ratios are a) 60%, b) 50%, c) 40%, d) 30%, e) 20% and f) 10%. Therefore, based on good shape memory effect, a certain LM-SMF could be molded by various shape and kept the deformation.

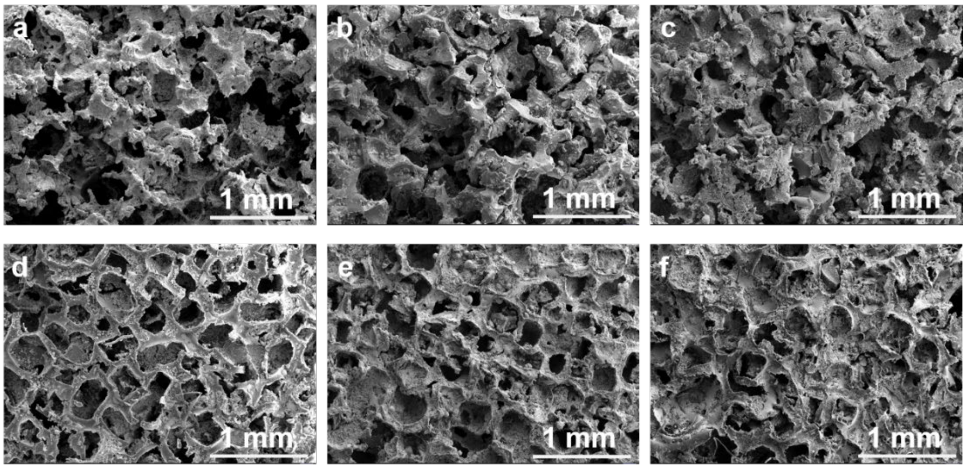

Figure S19. SEM images of LM-SMF (50wt% LM content) with various compressive ratio: a) 10%, b) 20%, c) 30%, d) 40%, e) 50% and f) 60%. As exhibited in SEM images, compressing leads to a compact porous structure and closer skeleton structure, which causes constant bulk LM connecting together and filling the pores.

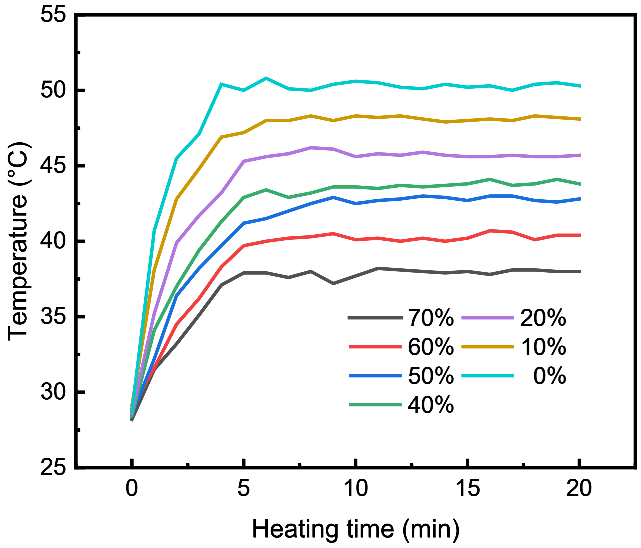

Figure S20. Surface temperature of LM-SMF with different compressive ratio on same heating film. As a result, surface temperature of LM-SMF becomes stable after heating about 5 min. Meantime, by increasing compressive ratio, the stable temperature increases from 38 °C to 50 °C, indicating that the thermal transmission capacity of LM-SMF improves with the increasing of compressive strain.

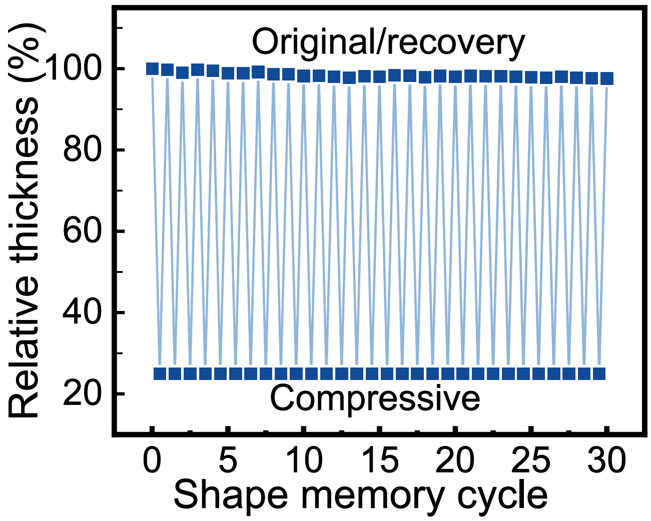

Figure S21. Shape memory cycle test of LM-SMF (50 wt% LM content). The shape memory property of LM-SMF maintains well even after 30 cycles of shape memory tests, and shape memory recovery ratio still reaches to 98%.

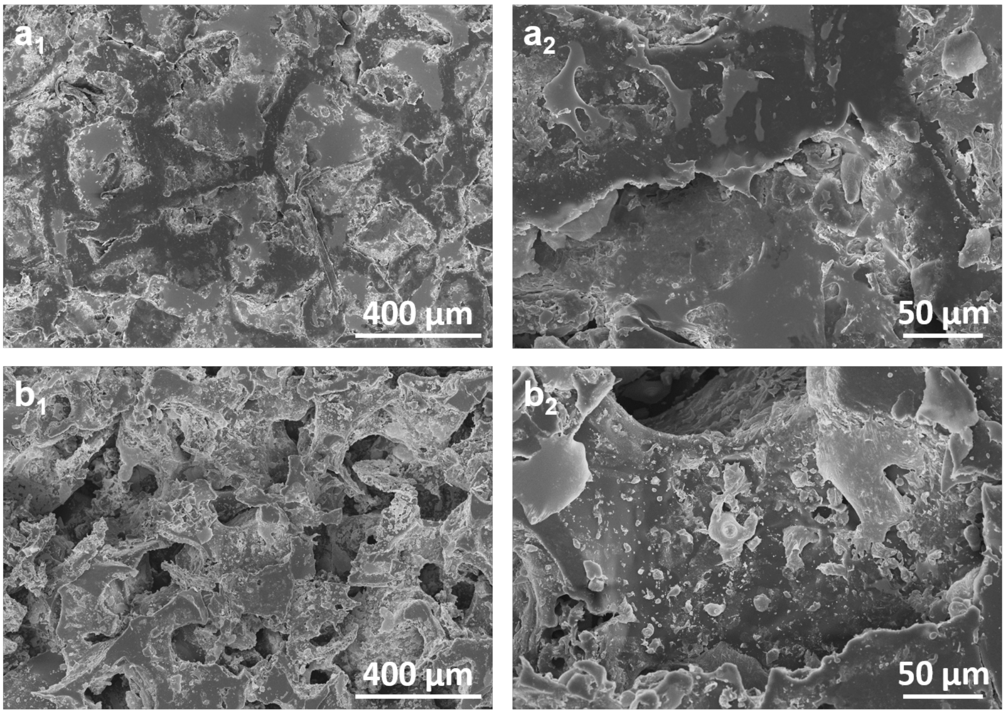

Figure S22. SEM images of a) compress and b) recovery LM-SMF at 30 shape memory cycle. One can see that after 30 cycles LM can still gather in compressive state and separate to bigger LM particles after recovery, meaning that LM can stably distribute on SMF skeleton after 30 cycles.

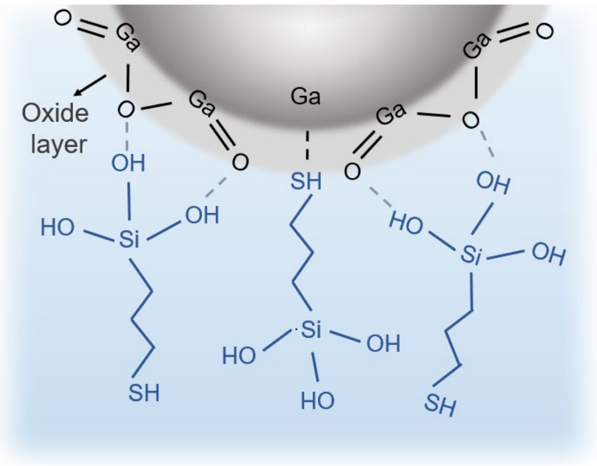

Figure S23. Schematic illustration shows the metal coordination, hydrogen bonds between MPTES and the surface of Gallium particles. There are two interactions between LM particles and MPTES, including metal coordination bond between Ga and -SH of MPTES[2] and hydrogen bonds between -OH of MPTES and oxide layer (Ga_2O_3),[9-11] which make MPTES combine with LM particles more stably.

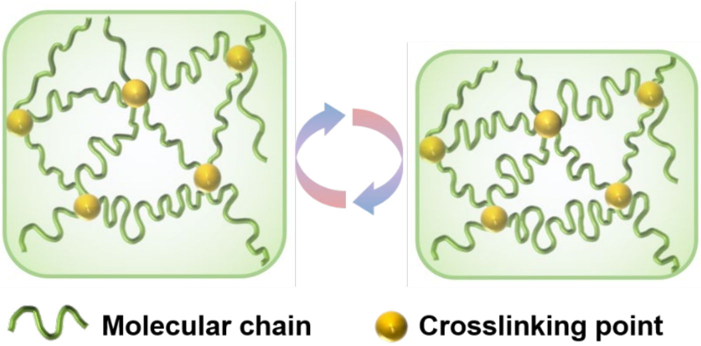

Figure S24. The molecular chain of LM-SMF skeleton changes in the shape memory progress.

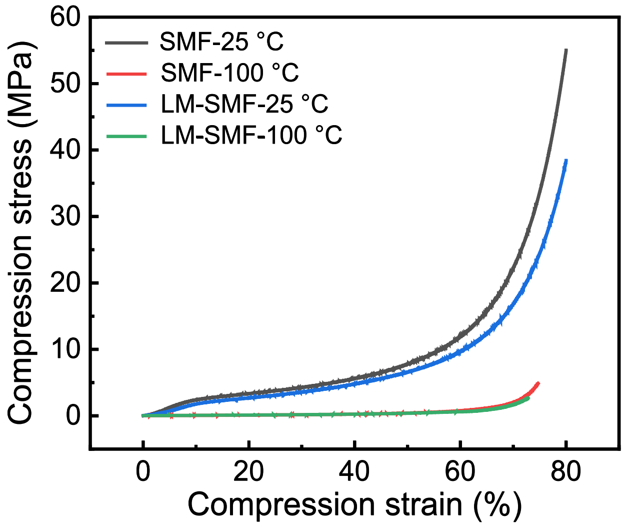

Figure S25. Compression stress-strain curves of SMF and LM-SMF (at 25 °C and 100 °C). As shown in bending test results, SMF and LM-SMF show the variable compression stress under 25 °C (below T_g) and 100 °C (above T_g). At 25 °C, LM-SMF exhibits high mechanical strength, and the compression stress can reach 38.2 MPa. At 100 °C, SMF and LM-SMF have smaller mechanical strength, and the compression stress is only about 2.8 MPa. In addition, the modification of LM does not affect the mechanical property obviously.

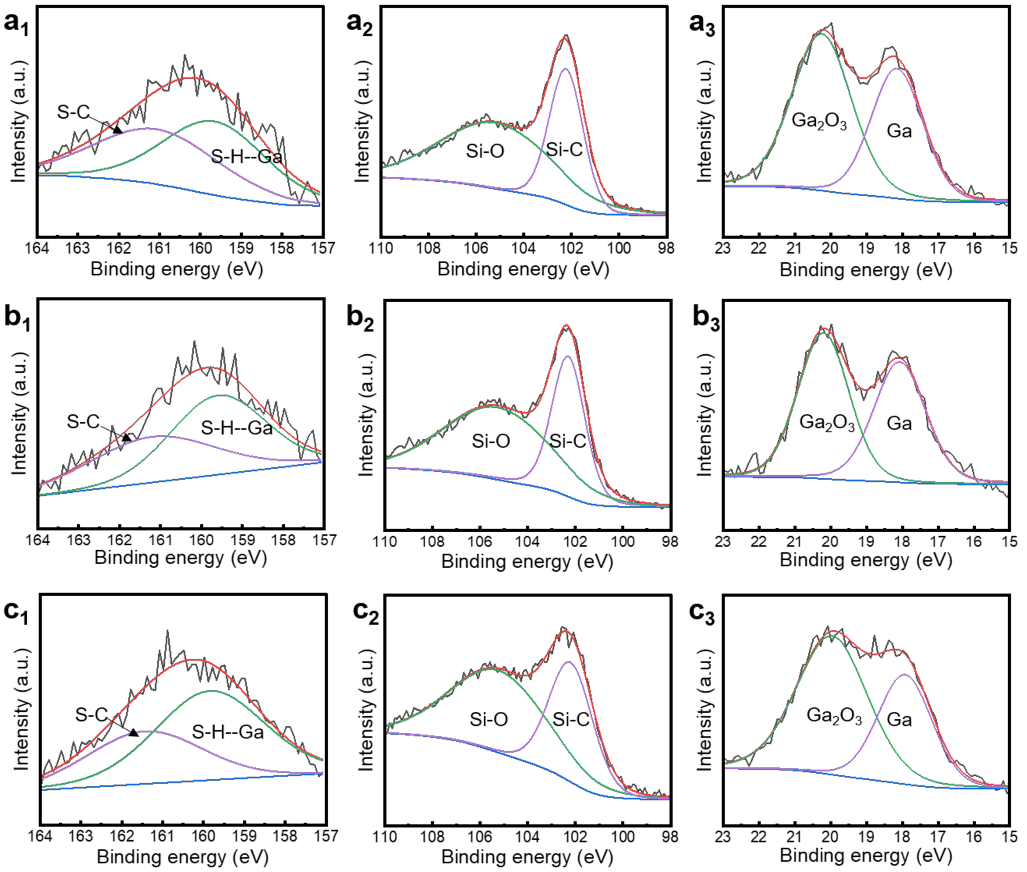

Figure S26. XPS high-resolution spectrum of a) original, b) compressed, and c) recovery LM-SMF indicates that there is no chemical reaction in the deformation progress of LM-SMF, and the chemical structure of foam is stable in the electrical/thermal controlling process.

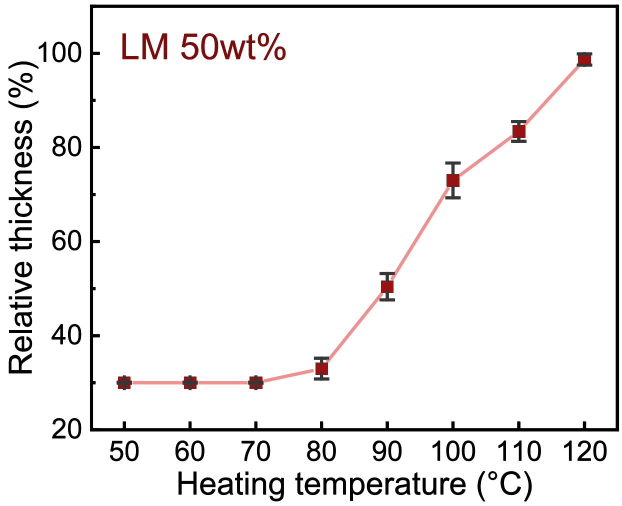

Figure S27. The relative thickness of LM-SMF changes with heating temperature. Herein, heating relied on heating film and only the bottom of sample can contact the heating source. As a result, because of shape memory effect of LM-SMF, when temperature reaches 80 °C, the foam begins to recovery. At 80 °C, relative thickness just has 3% increase. Then, the relative thickness increases with increasing the heating temperature further and recovers completely at 120 °C. Based on the above-mentioned shape recovery of LM-SMF, it is possible to design a self-feedback/-warning integrated smart switch according to the temperature-responsive relative thickness.

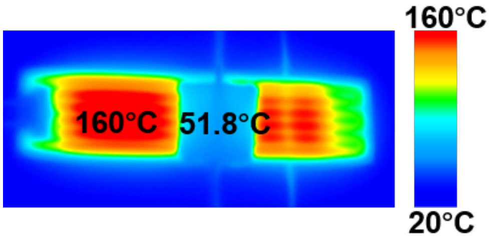

Figure S28. IR images of self-feedback/-warning integrated smart switch at overheated state (simulating the accident). In this situation, the temperature of heating film is too high (about 160 °C), meaning that the circuit is in abnormal working state.

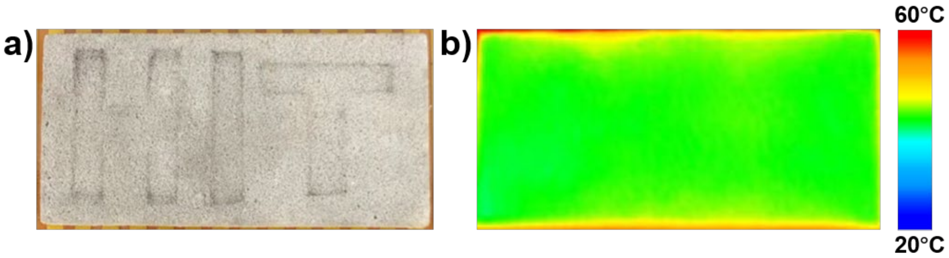

Figure S29. Photograph and IR image of recovery LM-SMF with "HIT" pressure marks, proving that restored indentation has no effect on the infrared stealth ability.

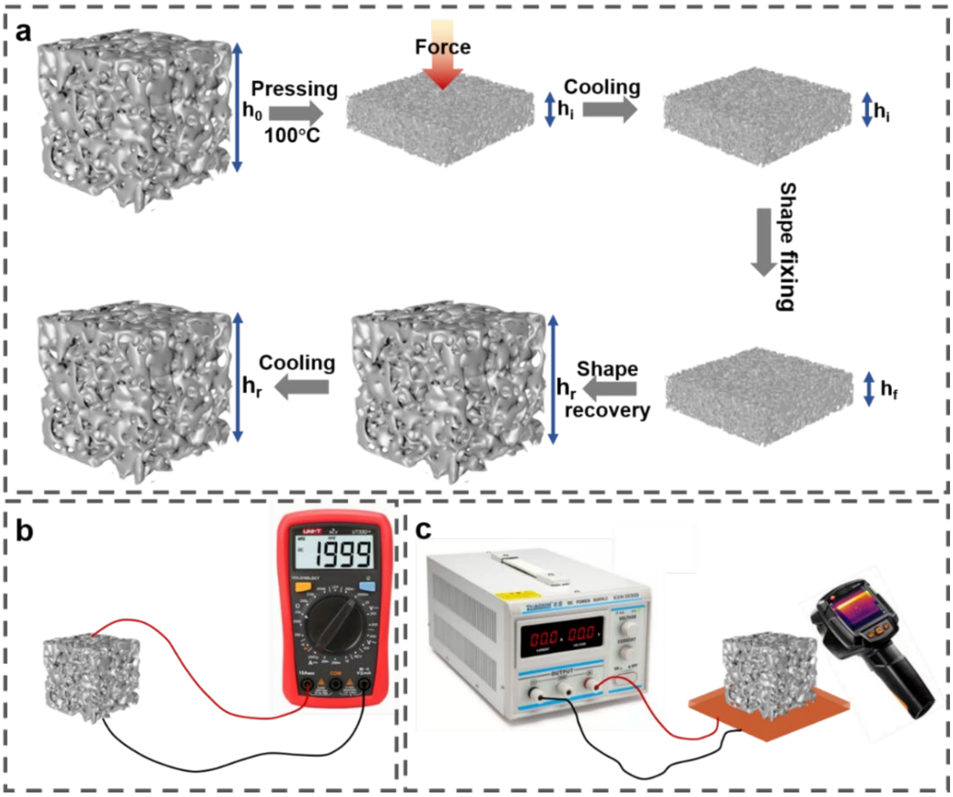

Figure S30. Schematic diagrams of test methods. a) Shape memory process, b) resistance and c) IR imaging.

References

[1] Y. M. Xin, H. Peng, J. Xu, J. Y. Zhang. *Adv. Funct. Mater.* **2019**, 29, 1808989.

[2] L. Mou, J. Qi, L. X. Tang, R. H. Dong, Y. Xia, Y. Gao, X. Y. Jiang. *Small* **2020**, 16, 2005336.

[3] Y. H. Wu, Z. Y. Fang, W. J. Wu, K. Q. Li, M. H. Lin, Z. M. Dang. *Adv. Eng. Mater.* **2021**, 23, 2100372.

[4] S. Y. Liao, X. Y. Wang, X. M. Li, Y. J. Wan, T. Zhao, Y. G. Hu, P. L. Zhu, R. sun, C.P. Wong. *Chem. Eng. J.* **2021**, 422, 129962.

[5] Z. G. Heng, Z. Zeng, Y. Chen, H. W. Zou, M. Liang. *J. Polym. Res.* **2016**, 22, 203.

[6] Y. Q. Ling, J. M. Luo, Z. G. Heng, Y. Chen, H. W. Zou, M. Liang. *React. Funct. Polym.* **2021**, 157, 104742.

[7] W. Zhang, J. Z. Ou, S. Y. Tang, V. Sivan, D. D. Yao, K. Latham, K. Khoshmanesh, A. Mitchell, A. P. O'Mullane, K. Kalantar-zadeh. *Adv. Funct. Mater.* **2014**, 24, 3799-3807.

[8] X. J. Wang, M. Y. Zhao, L. Zhang, K. Li, D. Wang, L. Zhang, A. M. Zhang, Y. Xu. *Chem. Eng. J.* **2022**, 431, 133965.

[9] M. A. Rahim, F. Centurion, J. Han, R. Abbasi, M. Mayyas, J. Sun, M. J. Christoe, D. Esrafilzadeh, F. M. Allioux, M. B. Ghasemian, J. Yang, J. Tang, T. Daeneke, S. Mettu, J. Zhang, M. H. Uddin, R. Jalili, K. Kalantar-Zadeh. *Adv. Funct. Mater.* **2021**, 31, 2007336.

[10] X. P. Hao, C. Y. Li, C. W. Zhang, M. Du, Z. Ying, Q. Zheng, Z. L. Wu. *Adv. Funct. Mater.* **2021**, 31, 2105481.

[11] X. Wang, M. Zhao, L. Zhang, K. Li, D. Wang, L. Zhang, A. Zhang, Y. Xu. *Chem. Eng. J.* **2022**, 431, 133965.
